# The differences between broad bean koji fermented in laboratory and factory conditions by an efficient *Aspergillus oryzae*

**DOI:** 10.3389/fmicb.2023.1139406

**Published:** 2023-03-22

**Authors:** Heng Li, Zhen-Ming Lu, Wei-Qin Deng, Qi-Sheng Zhang, Gong Chen, Qi Li, Zheng-Hong Xu, Yan-He Ma

**Affiliations:** ^1^Key Laboratory of Industrial Biotechnology of Ministry of Education, School of Biotechnology, Jiangnan University, Wuxi, China; ^2^Sichuan Food Fermentation Industry Research and Design Institute Co., Ltd., Chengdu, China; ^3^National Engineering Research Center of Cereal Fermentation and Food Biomanufacturing, Jiangnan University, Wuxi, China; ^4^Tianjin Institute of Industrial Biotechnology, Chinese Academy of Sciences, Tianjin, China

**Keywords:** *Aspergillus oryzae*, broad bean paste-meju, fermentation condition, koji, fermentation starter

## Abstract

Broad bean paste-meju was fermented by a mixture of broad bean koji and saline; koji fermentation is an essential process for the production of broad bean paste-meju. *Aspergillus oryzae* was the most widely used in sauce fermentation. The purpose of this study was to research the factory adaptability of the highly efficient *A. oryzae* PNM003 and further evaluate the effect of fermentation conditions and fermentation strains on koji. *A. oryzae* PNM003 was compared with the widely used strain HN 3.042 not only in the laboratory but also in factory conditions (large scale). Results showed that the koji made with the same starter in the factory had a greater amount of fungi than that in the laboratory. Bacteria and yeast levels in HN_L koji were higher than in PN_L koji. As for fungi constitution, almost only *Aspergillus* survived in the end through the microorganism self-purification process during koji fermentation. As for the bacterial constitution, koji was grouped by fermentation conditions instead of fermentation starter. PN koji had higher protease activity and a higher content of total acids, amino acid nitrogen, amino acids, and organic acids in the laboratory conditions. Nevertheless, in factory conditions, PN koji and HN koji had similar indexes. As for volatile flavor compounds, koji made with the two starters in the same condition was grouped together. As for the same starter, there were more flavor compounds metabolized in the factory condition than in the laboratory condition, especially esters and alcohols. The results showed PN was a highly efficient strain to ferment koji, but the advantages were expressed more remarkably in laboratory conditions. In brief, the fermented condition had a greater influence than the fermentation starter for broad bean koji.

## 1. Introduction

Chinese broad bean chili paste (also called *Pixian douban* or *Doubanjiang*) is extensively used in Chinese cuisine and is becoming increasingly popular in the whole world due to its nutritional benefits, bright red color, and unique pleasant aroma (Li X. et al., 2017). It was mixed with chili paste and broad bean paste-meju, then fermented in an open environment through stirring, sunlight exposure, and night dew until ripe. Broad bean paste-meju is a starter used for the fermentation of Chinese broad bean chili paste that contributes most flavors (Zhang et al., 2020a,b). Broad bean paste-meju is also widely used as a condiment and food coloring ingredient in Asian countries (Zhu et al., 2017). Broad bean paste-meju was fermented by mixing broad bean koji (also called Meibanzi) with saline water. Broad bean koji was made as follows. Shelled broad beans were softened by dipping them in hot water, cooled with cold water, blended with 17% (w/w) of wheat flour, and cultured in an environment with a certain temperature and humidity for 48–72 h.

During the koji making of traditional fermented condiments, microorganisms from the starter and environment reproduce and metabolize rapidly. As for broad bean paste-meju, *Bacillus amyloliquefaciens, Bacillus licheniformis, Bacillus subtilis, Aspergillus oryzae, Candida versatilis*, and *Zygosaccharomyces rouxii* were the main microorganisms that were derived from broad bean koji (Lu et al., 2021). Similarly, it was reported that more than 30 species of microorganisms in the genera *Aspergillus, Candida, Meyerozyma*, and *Lecanicillium* have existed in *douchi* koji inoculated with *Aspergillus* (He et al., 2019). *Weissella, Staphylococcus, Aspergillus, Candida*, and more than 30 other species were identified in Chinese soy sauce koji, which was inoculated with *Aspergillus* spores (Yan et al., 2013). Besides, microorganisms produce various enzymes, such as protease, peptidase, amylase, cellulose, and lipase in order to obtain nutrients necessary for growth by degrading cereals consisting of complex tissues (Yamashita, 2021). Amylases are predominant hydrolase enzymes that hydrolyze glycosidic bonds present in starch molecules and produce dextrins and oligosaccharides (Sundarram and Murthy, 2014). *B. amyloliquefaciens* and *A. oryzae* may be the main sources of amylases (Deng et al., 2020).

As we all know, enzymes were secreted by proliferating microorganisms during the koji fermentation period. The microorganisms of the fermentation starter were the most remarkable genus, and other strains were derived from the environment during fermentation. Thus, it is important to choose the appropriate strain as a starter for koji fermentation. *A. oryzae* cultures were able to give a fermented cake with a low pH, high amylase, acid protease, and carboxypeptidase enzyme activities, and low levels of free sugars; thus, it was used in fermented sauces such as soy sauce (Yokotsuka, 1983). *Aspergillus oryzae* HN 3.042, originally isolated by the Shanghai Niangzao Institute (Shanghai, China), is now commercially available for soy sauce production in China (Xu et al., 2009). *A. oryzae* RIB40 is an important strain widely used for soy sauce fermentation in Japan (Masuo et al., 2005; Zhao et al., 2013). Moreover, there have been numerous studies about screening efficient *Aspergillus* to ferment different kinds of sauces (Liu et al., 2015; Yu, 2021). *A. oryzae* HN 3.042 can not only grow well but also metabolize proteases and amylases in different environments. Hence, it was widely used in China not only for soy sauce production but also for other sauces, including broad bean paste-meju. However, the fermentation process of broad bean paste-meju is quite different from that of soybean sauce. Broad bean, the raw material for broad bean paste-meju fermentation, contains fewer proteins and more starch as compared with soybean. Moreover, the soybeans are steamed for several hours before the soybean sauce fermentation begins, while broad beans are only blanched for a few minutes in broad bean paste-meju fermentation. This way of pretreating raw materials results in the main producing companies in the Pixian area could not get the ideal broad bean koji (*Aspergillus* total number and enzyme activity were not enough), which led to the low fermentation efficiency of broad bean paste-meju (Lu et al., 2021). To date, the effect of different *A. oryzae* strains on the fermentation of broad bean koji is still lacking, so it is meaningful to screen and domesticate special strains for the distinctive fermentation of broad bean paste-meju.

Broad bean koji is fermented with the traditional processing method in an open environment, hence an abundance of microorganisms are involved in the fermentation process. Li Z. et al. (2017) investigated the microbial community and aroma compounds of traditionally fermented PBP (made by farmhouses) relative to those of industrially fermented PBP. Results showed several species of yeasts, molds, bacteria, and lactic acid bacteria, and 28 unique aroma substances were only detected in the traditional fermentation of PBP. Wang et al. (2017) proved that Daqu was the main source of strict aerobes and facultative aerobes, which took up more than 74% of prokaryotic communities in fermented grain. Pang et al. (2018) and Wang et al. (2018) emphasized that environmental microbiota were an important source of fermentation microbiota. Environmental microbiota can drive the stability of the liquor fermentation ecosystem and are highly relevant to the microbial succession and metabolic profiles during liquor fermentation. All the references indicated the fermentation environment and production scale can make significant differences in the mature koji. Most of the studies about highly efficient strains were researched in laboratory conditions (Ao et al., 2018; Ding et al., 2019; Yee et al., 2021; Hu et al., 2022), and their performance in the actual production environment of the factory remains unclear.

In this study, an efficient strain *A. oryzae* PNM003 that was previously screened by our research group was used to initiate the fermentation of broad bean koji, and its fermentation efficiency was compared with *A. oryzae* HN 3.042. Different from previous studies, the effects of these two strains on the fermentation of broad bean koji were compared not only under laboratory conditions but also under large-scale factory conditions, respectively. For the purpose of verification, the highly efficient *A. oryzae* strain PNM003, which was fit for fermenting broad bean koji, further gets superior broad bean paste-meju and Chinese broad bean chili paste.

## 2. Materials and methods

### 2.1. Materials

*A. oryzae* PNM003, which has a strong enzyme production capacity, was used in this study. It was preserved in the Guangdong Microbial Culture Collection Center in China, with the deposit number GDMCC 60615. Spore powder of *A. oryzae* HN 3.042 was bought from Ji Ning Yuyuan Biological Technology Co., Ltd. (Shandong, China).

Broad beans and flour were bought from Chongning Food Co., Ltd. (Chengdu, China). Chemicals like acetate solution and trichloroacetic acid were analytically pure and purchased from Kelong Chemical Co., Ltd. (Chengdu, China). Standard substances were obtained from Aladdin (Shanghai, China). Primers in this study were purchased from Sangon Biotechnology Co., Ltd. (Shanghai, China).

### 2.2. Methods

#### 2.2.1. Characterization of *A. oryzae* strains

Strain PNM003 (PN) was inoculated in soybean juice medium plates, cultivated at 28°C for 72 h. Then its colonial morphology and microstructure were observed and recorded. Furthermore, ITS sequences were determined for species identification. To identify it more accurately, strain PN was inoculated into AFPA medium plates and cultivated at 28°C for 72 h to observe the color reaction. In addition, for security verification, strain PNM003 was inoculated in a PD liquid medium, cultivated at 28°C for 180 h, and then its AFB1 content was detected with ELISA.

To detect the enzyme production capacity, strain PN was inoculated in a bran medium, cultivated at 28°C for 72 h, and the bran was shaken at irregular intervals to get more oxygen. The α-amylase activity was detected following the method of Simair et al. (2017), and the protease was determined referring to the experimental method of SB/T 10317-1999.

*A. oryzae* HN 3.042 (HN) spore powder was bought from Ji Ning Yuyuan Biological Technology Co., Ltd. PN spore powder was obtained by inoculating PN to bran medium. The bran was shaken timelessly by cultivating at 28°C for 72 h. Next, the spore-mixed culture was dried at 50°C for ~48 h, and then the spores were separated by an oscillating screen with an 80-mesh sieve.

#### 2.2.2. Broad bean koji preparation

Shelled broad beans were softened by dipping them in boiled water for 3 min, then cooling them with cold water, and draining off the water. Next, they were mixed with 15% wheat flour, and the mixture was inoculated with HN spore powder and PN spore powder, respectively. Then, it was fermented for 48 h at 30°C. Koji was turned over after incubating for 12 h to loosen the koji and release heat. There were two groups: kojis were made in laboratory and factory conditions (on a large scale) to detect the effect of the environment on the koji quality. The lab groups contained 2 kg of dry broad beans for each sample, and three repetitions were made for each fermentation starter. The kojis were cultured in a constant temperature and humidity incubator. For factory groups, each fermentation bar tank was made with 2 tons of broad bean, three repetitions were made for each fermentation starter, and the temperature was controlled by a temperature measuring device and a blower device. Koji making process in the laboratory and factory is shown in Supplementary Figure S1. Samples were taken regularly, and all the samples were freeze-dried before index detection.

#### 2.2.3. Physicochemical analysis

Total acidity (TA) was measured by 0.05 mol/L NaOH, yielding a titration endpoint at pH 8.2, as stipulated by the national standard of China (GB/T 12456-2008). The acid amino nitrogen (AAN) was quantified by the titration method according to the national standard of China (GB 5009.235-2016).

##### 2.2.3.1. Flavor components detection

OAs and FAAs were analyzed using HPLC (Agilent, 1260 Infinity, GER) with a DAD detector. OAs detection: Briefly, 1 g of broad bean paste-meju was taken into a 50-ml plastic tube and vortex-mixed with 10 ml of ddH_2_O; ultrasonic extraction was performed for 30 min (100%, 30°C). Then the mixture was centrifuged at 8,000 r/min for 10 min, and the supernatant was filtered for detection. The detection was performed on a column sepaxme-h (NP) 7.8 × 300 mm (US) at 55°C, separated by a mobile phase consisting of 0.02 mol/L H_2_SO_4_ at a flow rate of 0.6 ml/min, and the detection wavelength was 210 nm.

Volatile flavor components and FAAs were detected using the method described in our previous research (Li et al., 2023). FAAs were detected with HPLC (Agilent 1260 Infinity II, GER), performed on a column (4.6 × 250 mm, 5 μm) (Ultimate Amino Acid, Welch, USA) at 40°C. Volatile flavor components were detected by a Shimadzu gas chromatograph (Shimadzu, Kyoto, Japan) with a DB-WAX column (Agilent, Santa Clara, USA).

##### 2.2.3.2. cDNA extraction and Illumina Miseq sequencing

A quantity of 10 g of sample was mixed with 90-ml of sterile water and filtered through sterile gauze to remove large particles. After centrifuging at 12,000 rpm for 10 min at 4°C, the pellets were used for genomic DNA extraction according to the instructions of the producer who supplied DNA extraction test kits (omega, America). DNA quality and quantity were assessed using a Nanodrop spectrophotometer (Thermo Fisher Scientific) and diluted to 10 ng/μl for downstream use. Universal primers 338F/806R were used to amplify bacterial 16S rRNA for Miseq sequencing, respectively. The TransGen AP221-02 kit with TransStart Fastpfu DNA Polymerase (TransGen Biotech, Beijing, China) was applied in PCR reactions and then visualized by 2% (w/v, TBE buffer) agarose gel electrophoresis at room temperature. Electrophoretic bands with the correct size were excised and purified by the AxyPrepDNA Gel Recovery kit (Axygen Scientific, Inc., CA, USA) and then quantified using a QuantiFluo-ST Blue Fluorescent Quantitative System (Promega, Madison, Wisconsin, USA) after being eluted by Tris-HCl. The TruSeqTM DNA Sample Prep kit was provided for preparing and sequencing the samples according to the manufacturer's instructions, and the amplified product was then sequenced by the Illumina Miseq sequencer (Illumina, Inc., CA, USA). The sequence data were analyzed according to Lu et al. (2020)'s description.

##### 2.2.3.3. Statistical analysis

All tests were carried out in triplicate. All the indexes of the samples were shown as dry basis weight in this article. Data were presented as means ± standard deviation (SD). Excel 2019 and Origin 2022 were used to analyze the data. The heatmap graph was dealt with TB tools; PCA was analyzed using SIMCA 14. The fragrance of volatile flavor compounds was referred to the database The Good Scents Company Categories of products. Analysis of Pearson's correlation between microbial communities and flavors was conducted using Spass 22, in which the relationships of whose *P*-values below 0.05 (*P* < 0.05) were visualized using the Cytoscape software (version 3.9.1, https://cytoscape.org/download.html). Additionally, all experiments were conducted in triplicate, and the statistical data is shown as the mean value ± standard deviation.

## 3. Results and discussion

### 3.1. Characterization of strain PN

**Figure 1:**
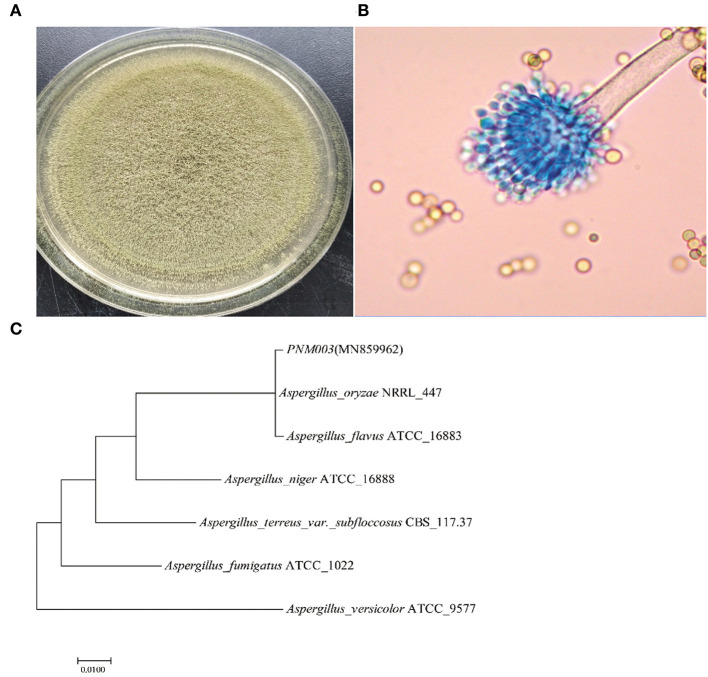
**(A–C)** Morphological characteristic and phylogenetic tree of *A. oryzae* PNM003.

Colony and microstructure photos of strain PN are shown in Figures 1A, B. Strain PN has yellowish-green, short mycelium after being cultured on a PDA plate. Microscopic observation results showed that the sporophore wall was thin and smooth. Conidial heads were typically radiate or biseriate, with some heads having phialides borne directly on the vesicle (uniseriate). Conidiophore stipes were hyaline and coarsely roughened. Conidia were subglobose to globose. There was no sclerotium observed. Strain PN was most similar to *A. oryzae* according to the *Aspergillus* identification method reported by Bai et al. (2018), depending on colony morphology and micromorphology.

The phylogenetic tree is shown in Figure 1C. PN was clustered with *A. oryzae* and *A. flavus*. It was reported that *A. oryzae* and *A. flavus* were quite similar in ITS rRNA gene sequences, thus other methods were necessary for comprehensively distinguishing the two species. Quantitative analysis of AFB1 results showed that the reverse side of the colony was yellowish-brown after being cultured in AFPA medium for 3 days, indicating strain PNM003 did not produce AFB1. Moreover, AFB1 was not detected in the PN fermentation broth, further testifying that it would not cause AFB1 contamination. It further identified that PN was *A. oryzae*. Moreover, strain PN was a strong enzyme producer. Enzyme activity test demonstrated that PN can produce 2,490.19 ± 51.92 U/g α-amylase, 2,215.82 ± 30.081 U/g neutral protease, and 1,349.32 ± 28.39 U/g acid protease after cultivating in bran medium for 3 days.

### 3.2. SEM images of broad bean and broad bean koji

Supplementary Figure S2 shows the SEM images of the broad bean and broad bean koji. Starch and protein were the main components of broad beans. The shapes of the starch granules were long elliptical and spheroidal (Hao et al., 2021). The protein matrix seems to have a spongy structure covered on the surface (El-Shimi et al., 1980). Starch granules in cells were tightly embedded in protein network structures. Compared with dried broad beans, starch granules of blanched broad beans swelled slightly. Some proteins of the blanched broad beans denatured and wrapped around starch granules (Ding et al., 2011). The samples made in fac denatured more. Compared with blanched broad beans, starch granules of broad bean kojis were shrinking, and there was a lot of moisture lost during the koji fermenting process. Some proteins of kojis around starches were broken down by enzymatic effect, they changed from slices covering the starch granules at first to some particles attached to the starch granules.

### 3.3. Broad bean koji fermentation ability evaluation

#### 3.3.1. Diversity of microbial population

**Figure 2:**
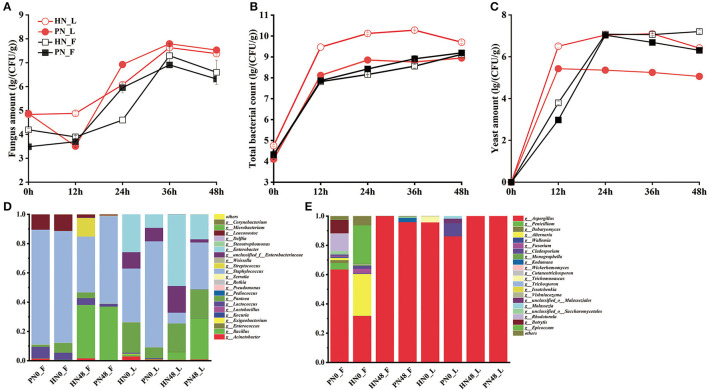
**(A–E)** Microbial community composition of koji fermented with different starters and different conditions.

The diversity of the microbial population was shown in Figures 2A–D. The succession of the microbial community in koji made with PN and HN was similar. In general, the total number of bacteria increased rapidly in the first 12 h, then tended to be stable. The bacteria count for koji made by HN in the laboratory was higher than PN, indicating koji inoculated with PN contaminated with fewer competitors. As for yeast, it reached about 1 × 10^7^ CFU/g in koji made in the factory with both starters. In the laboratory conditions, the yeast amount of koji fermented by the HN starter was more than the PN starter. It may be because strain PN proliferates faster in laboratory conditions, and yeast growth was inhibited in the system. The fungus amount increased rapidly in the first 36 h, then declined slightly. The amount was similar in koji made with the two starters under the same condition, while the fungus amount of broad bean koji made with the same starter in the factory was slightly higher than in the laboratory condition.

#### 3.3.2. High throughput sequencing

As for fungal diversity, after removing the low-quality sequences, 381,705 reads were obtained. The taxonomic classification of ITS1_ITS2 gene sequences at the genus level is shown in Figure 2E. *Aspergillus* was absolutely the dominant fungus. The fungus was derived from material in the 0th day samples, and the fungus constitution was more abundant in fac koji. The fungus composition of koji (0th day) fermented by HN and PN starters was similar. For PN_F koji, *Aspergillus, Rhodotorula, Botrytis*, and *Epicoccum* were the largest group of fungi. For HN_F koji, *Aspergillus, Alternaria, Epicoccum*, and *Debaryomyces* were the largest group of fungi. There was relatively less fungal diversity for lab koji (0 h). For HN0_L, the relative abundance of *Aspergillus* reached 95.65% and the relative abundance of *Trichomonascus* was 4.21%. For PN0_L, the relative abundance of *Aspergillus* reached 86.11%, whereas the relative abundance of *Cladosporium* and *Malasseziale* was 9.00 and 4.90%, respectively. For the mature koji in the laboratory, there was almost only *Aspergillus*. As for mature koji in the factory, 99.99% of the fungus was *Aspergillus* in HN48_F, and 95.88% was *Aspergillus* in PN48_F. Moreover, 2.87% of *Kodamaea* and a lower amount of *Debaryomyces* and *Wickerhamomyces* were derived from the fermentation process. As we can see, the microbial diversity was more abundant in the 0th day samples. It can be speculated that *Aspergillus* was the absolutely dominant fungus, and there were almost no other molds detected. Supplementary Figures S3C, D show that the fungal community composition of broad bean kojis in this study was similar except for HN0_F and PN0_F.

As for bacterial diversity, after removing the low-quality sequences, a total of 361,085 reads of bacteria were obtained. These data indicated that the microbial diversities were significantly different among the koji made in the laboratory and factory with different starters. Taxonomic classification of bacterial 16S rRNA gene sequences at the genus level is shown in Figure 2D. Results showed that the koji-fermented environments made significant differences in bacterial composition, whereas the fermented starter made no difference in the bacteria constitution. For koji made in the factory environment, *Staphylococcus, Leuconostoc*, and *Lactococcus* were the main bacterial species for koji made by both starters at 0 h (material), and the content of *Staphylococcus* reached 78.44%. *Bacillus* content increased a lot during the koji fermentation period, and *Bacillus* and *Staphylococcus* were dominant genera for the mature koji. For lab koji, the bacterial constitution was similar for HN and PN koji. For 0-h samples, under laboratory conditions, *Staphylococcus, Pantoea*, and *Enterobacter* were dominant bacteria, and *Staphylococcus* and *Bacillus* content increased a lot during the koji-making process. It was consistent with previous reports that *Bacillus* was the dominant bacteria for douchi-koji (Chen et al., 2011). During the koji-making process, microorganisms proliferated and metabolized with heat release, causing the temperature in the koji to increase. Moreover, the oxygen and nutrient substances were not enough in the broad bean koji. Compared to other bacteria, *Bacillus* can resist adverse conditions, so its relative abundance increased the most during the koji-making process. Previous studies have shown that *Staphylococcus* might be the most abundant and widespread functional genus in broad bean paste. It was also reported that *Staphylococcus* played an important role in substrate alteration and flavor development in the broad bean paste fermentation and was usually used as the starting culture for fermentation (Mainar et al., 2016; Stavropoulou et al., 2018). Supplementary Figures S3A, B show that the bacteria of broad bean koji for the laboratory and factory conditions were located in the first quadrant and the third quadrant, respectively. Results showed that the bacterial community composition of broad bean koji was most affected by the environment microorganism rather than the leavening agent.

#### 3.3.3. Variation of enzyme activity and physicochemical factor

**Figure 3:**
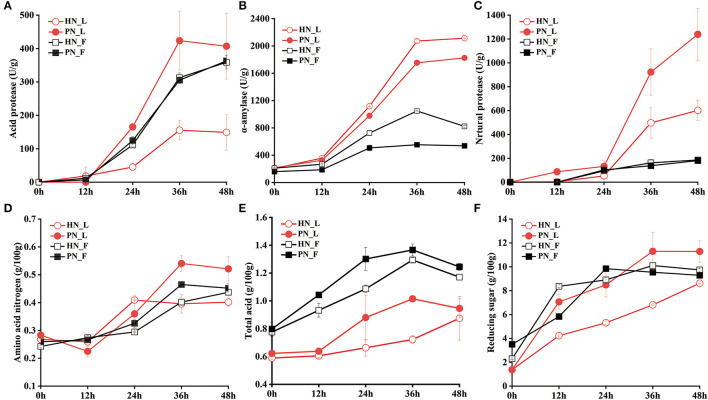
**(A–F)** Variation of enzyme activities and physicochemical factors of koji fermented with different starters and different conditions.

There was a significant difference between the activities of the enzymes for koji fermented by the two starters in the laboratory conditions. Figure 3B shows that α-amylase was mostly secreted from 12 h to 36 h; the amylase activity of HN koji was slightly higher than PN koji. Figures 3A, C shows that the protease activity (both acid protease and neutral protease) of PN koji were much higher than those of HN koji in the laboratory conditions. The acid protease activity of PN 48_L was 406.86 ± 98.05 U/g, while that of HN48_L was only 149.04 ± 53.36 U/g. Neutral protease activity of PN48_L was 1,239.17 ± 218.04 U/g, while HN 48_L was 601.35 ± 85.28 U/g at 48 h.

In the factory condition, amylase activity and protease activity for both PN koji and HN koji were much lower than in the laboratory condition. As for the amylase activity, HN koji was higher than PN koji. Figure 3B shows that the amylase activity for PN48_F and HN48_F were 2,113.25 ± 4.11 U/g and 1,824.99 ± 0.73 U/g, respectively, in the laboratory conditions, whereas it only reached 821.97 ± 11.09 U/g and 537.71 ± 11.06 U/g for lab koji at 48 h. There were no significant differences between the protease activities of koji made in the factory condition and those of the two starters. The protease activity of fac koji was slightly lower than the koji made in the laboratory conditions. Figures 3A, C show that the neutral protease and acid protease activities of fac mature koji were about 186 and 360 U/g, respectively.

Variation of physicochemical indexes is shown in Figures 3D–F. The total acids and amino acid nitrogen of koji made in the laboratory condition were much higher than in the factory condition. On the contrary, the total acids and amino acid nitrogen of koji made with PN were higher than those of the HN starter in the same condition. Reducing the sugar content increased gradually during the koji-making process. Reducing the sugar content of HN koji made in the laboratory conditions was the lowest. These results showed the macromolecular compositions were decomposed into micromolecular nutrients and flavor compounds through microorganism metabolization during the koji fermentation process. The results showed the a-amylase of HN_L activity was highest, but the reducing sugar content for it was the lowest, possibly due to the consumption by the highest amount of bacteria and yeast.

#### 3.3.4. Variation of amino acids

**Figure 4:**
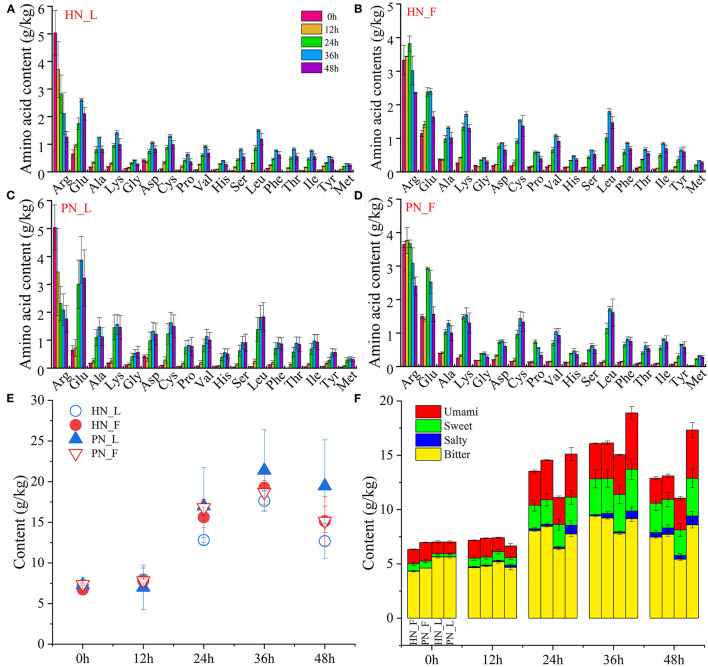
**(A–F)** Variation of amino acids of koji fermented with different starters and different conditions.

The total content of amino acids in koji was stable during the initial period. It increased rapidly from 12 h and reached its highest at 36 h. Figure 4E shows that the total content of amino acids in PN koji fermented in the laboratory condition was much higher than that of HN koji. There was no significant difference between this koji made in the factory condition with the two starters. It indicated that the fermentation environment had a greater influence than the starter. The variation trends of amino acid composition were consistent for the different starters at the same condition (Figures 4A–D). The variation trends of different amino acids are shown in Supplementary Figure S4. A total of 17 kinds of amino acids were detected in this study. Supplementary Figure S4 shows that all the amino acid contents increased gradually during the koji-making process except arginine. The concentrations of arginine and glutamic acid were the highest. Arginine was originally presented in raw material, with an amount of ~5.03 g/kg at 0 h, and then reduced to 1.77 and 1.26 g/kg for fac koji. It was ~3.33 and 3.64 g/kg at 0 h and then reduced to 2.36 and 2.41 g/kg for lab koji. Arginine occupied a significant role in nutrition due to its multiple and sometimes unique physiological and pharmacological actions. Although in unstressed animals and humans, it might be classified as a dietary semi-dispensable amino acid, arginine becomes an indispensable dietary amino acid in clinically important states such as starvation, injury, or stress (Barbul, 1986). Arginine can be converted to citrulline (Hill et al., 2003), ornithine (Poolman et al., 1987), and proline (Van Hoof et al., 2007). Otherwise, it may further convert to a biogenic amine. Arginine and L-ornithine were precursor amino acids of putrescine, which may explain why they declined precipitously during the koji-making process. The concentration of glutamic acid increased at first, then degraded over 36 h in all groups. Glutamic acid can be accumulated through protein or polypeptide degradation; it can also be yielded from glutamine catalyzed by glutaminase. Plenty of protease and peptidase could be secreted by *Aspergillus* during the koji-making stage (Zhang et al., 2020a,b); hence, other amino acid concentrations increased gradually.

Figures 4E, F shows the total content and classification of amino acids depending on taste. There were bitter, umami amino acids detected in the raw materials (0 and 12 h samples). Amino acids increased to a high content at 24 h, and all kinds of amino acids increased during the koji making process. Total amino acid contents of koji at 36 h were the highest and then decreased slightly at 48 h. Bitter amino acids were the main amino acids in koji; sweet and umami amino acids increased a lot during the koji-making process. Microorganisms and enzymes decompose macromolecular protein into micromolecular amino acids; hence, umami and sweet amino acids increased slightly during the koji-making process. The interesting result was the total content of amino acid and umami amino acid for lab koji made with strain PN was higher than HN. For amino acids constitution, there was no significant difference between the koji made in the factory with the two strains.

#### 3.3.5. Organic acid

**Figure 5:**
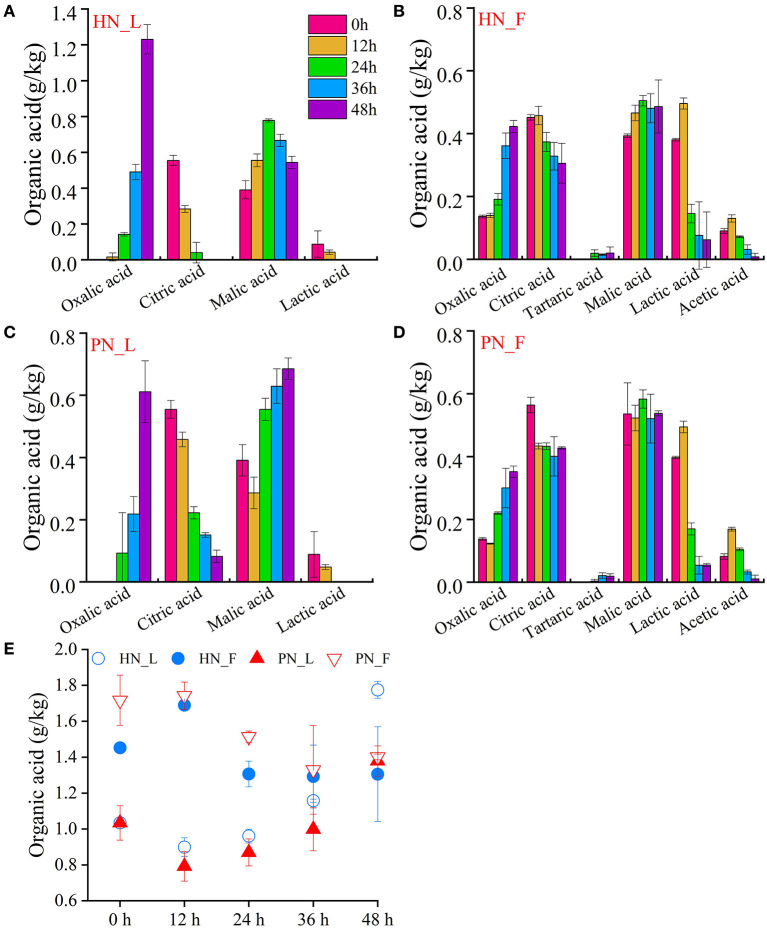
**(A–E)** Variation of organic acids of koji fermented with different starters and different conditions.

Six kinds of organic acids were detected in this study. Figure 5E shows the total content of organic acids during the koji-making process. In the laboratory condition, the organic acids content increased gradually, whereas, in the factory condition, the organic acids content was much higher than that of those in the laboratory condition at the beginning, but it decreased gradually during the koji-making process. This may be due to the distinction of materials such as broad bean, flour powder, and sources of water, which need further verification. Figure 5E shows that the total content of organic acids in PN koji fermented in the laboratory conditions was much higher than in HN koji, whereas there was no significant difference between the two kojis made in the factory condition. In addition, the constitution of organic acids was similar for koji made with the two starters in the same condition. The results indicated that the fermentation environments made a greater influence than the starters.

Organic acids constitution was similar for koji made in the same condition. Oxalic acid, citric acid, lactic acid, and malic acid were detected both in the laboratory and factory koji, whereas acetic acid and tartaric acid were only detected in factory koji with low concentrations. Supplementary Figure S5 shows that oxalic acid and malic acid were the main organic acid for broad beans; they increased during the fermentation process. The content of oxalic acid was the highest, and it was accumulated during the koji-making process. It was detected mostly in laboratory koji, especially HN koji. While other acid contents decreased gradually during the koji-making process. It may be because the content of acids used as nutrients by the microorganisms was more than they metabolized.

There was a small amount of lactic acid in the broad bean koji made in laboratory conditions, whereas it was more in factory koji. Lactic acid for HN0_F was 0.38 mg/kg, and it increased to 0.50 mg/kg and then decreased to 0.62 mg/kg at 48 h. For PN0_F, lactic acid was 0.40 mg/kg and then decreased to 0.05 mg/kg at 48 h. Furthermore, citric acid was detected at a high concentration in 0 h samples and then reduced during the koji-making process.

### 3.4. Volatile flavor profiles

**Figure 6:**
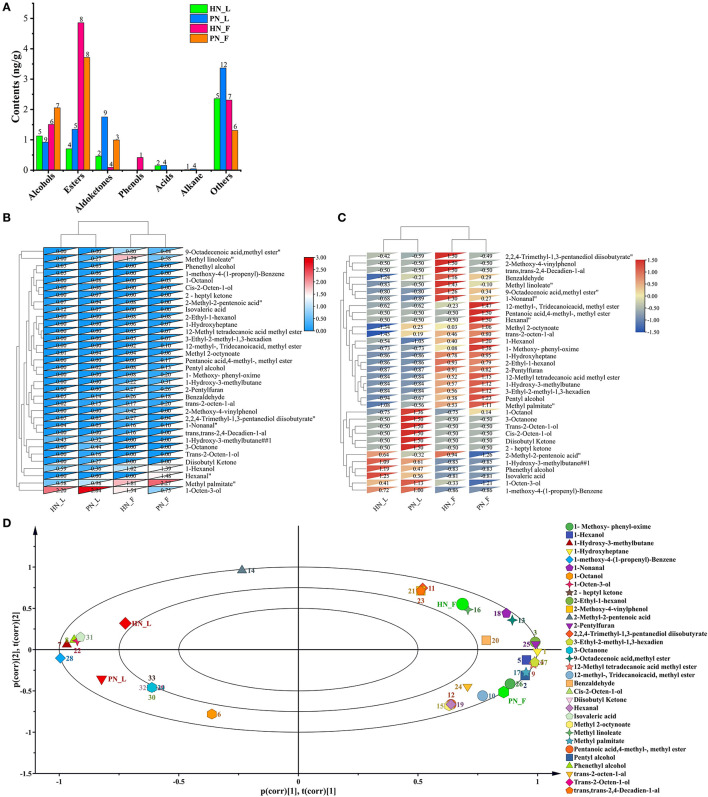
**(A–D)** Profiles of volatile flavor compounds of koji fermented with different starters and different conditions.

Koji was fermented only for 48 h. However, the fermentation condition was dry relatively, thus these macromolecular substances were not hydrolyzed totally, and hence not too many volatile flavor compounds were generated. However, the results showed that the clustering results were significant across all samples in this study. The main volatile flavor compounds of mature koji were alcohols and esters. There were 45 kinds of main flavors detected (Figure 6A). 1-Octen-3-ol (cucumber, earth, fat, floral, and mushroom), methyl palmitate (oily waxy fatty orris), and methyl linoleate (oily fatty woody) were the predominant volatile flavor of koji. Figures 6B, C shows that koji made with the two starters in the same condition was grouped together. The diversity and content of flavors for PN_L koji were more than for HN_L koji. Nevertheless, the flavor constitution of the two strains was similar in the factory condition. As for the same starter, there were more flavor compounds metabolized in the factory condition than in the laboratory condition, especially esters and alcohols. Results indicated that the fermented environment had a greater influence than the initial fermentation fungi.

PCA analysis (Figure 6D) showed that the four kinds of koji were located in four quadrants, principal component one contributed 63.9%, and principal component two contributed 20.9%. The distance between PN_F to HN_F and PN_L to HN_L were measured at the principal one level, indicating that the koji flavor was similar at the same fermentation condition. Results showed that koji made in the factory accumulated more volatile flavor compounds. 2,2,4-Trimethyl-1,3-pentanediol diisobutyrate, 2-methoxy-4-vinylphenol (spicy, burnt), (trans, trans-2,4-decadienal), and 2-methyl-2-pentenoic acid (sour, acidic, sweaty, berry, and fruit-like) were most correlated with HN_F group. 12-Methyl-tridecanoic acid, methyl ester, pentanoic acid, 4-methyl-methyl ester, and hexanal (grass, tallow, and fat) were most correlated with the PN_F group.

Comparing PN and HN koji made in the laboratory condition, 1-octanol (fresh orange rose odor), methyl 2-octynoate, trans-2-octen-1-al (fatty), cis-2-octen-1-ol (mild, sweet odor), diisobutyl ketone (mild, sweet odor), 2-heptyl ketone, and 3-octanone were most positively correlated with PN_L group. 1-Hydroxy-3-methylbutane, phenethyl alcohol (floral, sweet, rose, and bready), and isovaleric acid (sweaty, acidic, and rancid) were most positively correlated with the HN_L group. Results showed that in the factory conditions, flavor compounds of koji made with PN were more diverse and rich than HN koji. 2,2,4-Trimethyl-1, 3-pentanediol diisobutyrate, and 2-methyl-2-pentenoic acid (sour, acidic, sweaty, berry, and fruit-like) were most positively correlated with HN, but they were negatively correlated with PN. Whereas, pentanoic acid, 4-methyl-methyl ester, and hexanal (grass, tallow, and fat) were most positively correlated with PN, but they were negatively correlated with HN starter. Furthermore, pentyl alcohol, 1-hexanol (fatty and fruity), 2-ethyl-1-hexanol (mild, oily, and sweet), 1-hydroxy-3-methylbutane, 9-octadecenoic acid, methyl ester, trans-2-octen-1-al, 2-pentylfuran, and 3-ethyl-2-methyl-1,3-hexadiene were positively correlated with the two groups made in the factory (Figure 6D).

### 3.5. Correlations between microbial communities and flavors

**Figure 7:**
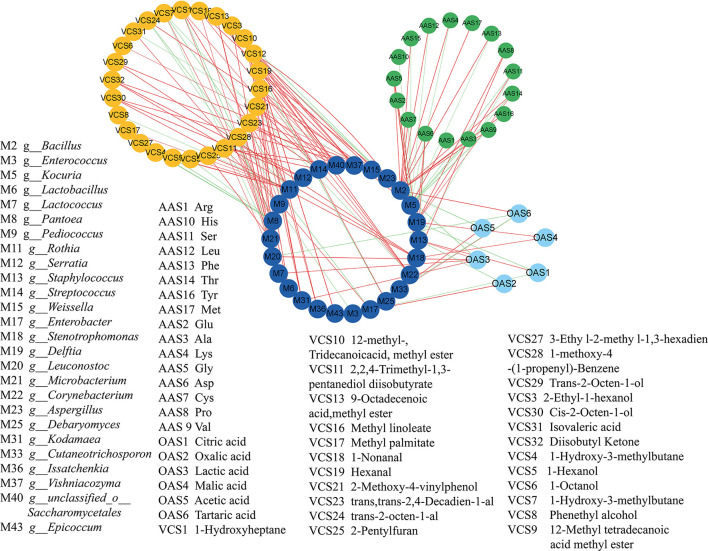
Correlation analyses between microorganisms and flavors by cytoscape. in koji fermented with different starters and under different conditions.

The relationship between the key microbial communities and the metabolites in the koji samples was analyzed using Pearson's correlation analysis. Close attention was paid to the microbial communities with correlation coefficients of >0.7 (*|r|*>0.7) and *P-*values of < 0.05 (*P* < 0.05). In total, 26 kinds of microbes were noted to be significantly related to flavors during koji fermentation (Figure 7), with the populations exhibiting close relationships with FAAs, OAs, and VCs, respectively.

The most abundant fungus *Aspergillus* was significantly positively correlated with pentanoic acid, 4-methyl-, methyl ester (fruity, sweet, banana, and pineapple), and hexanal (grass, tallow, and fat) with a correlation coefficient of 1.0 and negatively correlated with lactic acid and 2-methyl-, tridecanoicacid, methyl ester (*|r|* > 0.9). The abilities of *Aspergillus* to naturally accumulate high amounts of organic acids and to utilize a wide range of carbon sources have been widely reported (Yang et al., 2017). In soybean, the extracellular enzymes of *Aspergillus* hydrolyze soybean to different kinds of amino acids and sugars (Yong and Wood, 1977). It was reported that *Aspergillus* were positively correlated with protease and glucoamylase activity; however, *Staphylococcus* and *Zygosaccharomyces* were positively correlated with titratable acid, amino acid nitrogen, free amino acids, volatile flavor substances, and organic acids during broad bean fermentation process (Jia et al., 2020). *Kocuria* was negatively correlated with 12 kinds of OAs, but it was positively correlated with Arg. Furthermore, it was highly positively correlated with 1-nonanal, (9-octadecenoic acid and methyl ester), methyl linoleate (12-methyl, tridecanoic acid, and methyl ester), lactic acid, pentanoic acid (4-methyl and methyl ester) (*|r|* = 1.000), and hexanal (*|r|* = 1.000). The most abundant bacteria *g_Staphylococcus* was positively correlated with citric acid and lactic acid. It was consistent with the previous reports by Jia et al. (2021) *g_Bacillus* was positively correlated with 11 kinds of OAs and tartaric acid, trans-2-octen-1-al. Most of the *Bacillus* species can assimilate amino acids as carbon sources and energy (Turnbull et al., 1996). *g_Enterobacter* was positively correlated with tartaric acid and isovaleric acid. *g_Enterococcus* was positively correlated with oxalic acid. 12-methyl, tridecanoic acid, methyl ester was positive correlated with *g_Debaryomyces, g_Kodamaea, g_Vishniacozyma*, and so. The *Issatchenkia* can metabolize ester metabolites. 1-Nonanal (aldehydic citrus, cucumber, melon rindy potato, raw potato, oily nutty, and coconut) was highly positively correlated with *g_Corynebacterium* (*|r|* = 0.969), *g_Kocuria* (*|r|* = 0.986), *g_Lactococcus* (*|r|* = 0.989), and *g_Leuconostoc* (*|r|* = 0.969). Lactic acid was positively correlated with *g_Kodamaea, g_Lactococcus, g_Leuconostoc*, and *g_Staphylococcus*. Pentanoic acid, 4-methyl-, methyl ester (fruity, sweet, banana, and pineapple) was positively correlated with fungi such as *g_Aspergillus, g_Epicoccum, g_Issatchenkia*, and *g_Kodamaea*. Trans, trans-2,4-decadienal (fatty chicken, aldehydic green, and fried potato) was highly positively correlated with *g_Lactobacillus* (*|r|* = 0.997), *g_Leuconostoc* (*|r|* = 0.960), *g_Pediococcus* (*|r|* = 0.965), *g_Streptococcus* (*|r|* = 1.000), and *g_unclassified_o_Saccharomycetales* (*|r|* = 1.000). It was reported that *Lactobacillus, Lactococcus, Enterobacter*, and *Pseudoalteromonas* were significantly positively correlated with TTA (*P* < 0.05) (He et al., 2020). *Lactobacillus, Leuconostoc, Pediococcus, Psychrobacter, Pseudoalteromonas*, and *Rahnella* were significantly positively correlated with lactic acid (*P* < 0.05) (He et al., 2020). *Staphylococcus, Bacillus, Weissella, Aspergillus*, and *Zygosaccharomyces* were closely related to the formation of the flavor of BBP because of their abundance and their involvement in the flavor biosynthesis during fermentation (Jia et al., 2021). Tan et al. (2022) reported that the abundances of *Aspergillus, Kocuria, Enterococcus, Lactococcus*, and *Corynebacterium* were all positively correlated with the concentrations of all amino acids.

As far as we know, these microorganisms and enzymes are only metabolized to some extent through the koji fermentation process. The differences left by the microorganisms and enzymes in the koji may play a significant role during the next fermentation period of the broad bean paste-meju. We will further study the fermentation process and the quality of broad bean paste-meju fermented by the koji made in the laboratory and factory conditions by the efficient *A. oryzae* PN.

## 4. Conclusion

The results showed that strain PN was a highly efficient strain for fermenting koji. In this study, koji was grouped by fermentation conditions instead of fermentation starter for bacteria constitution. PN koji had higher protease activity and a higher content of physicochemical indexes in the laboratory conditions. To our surprise, while tested on a factory large scale, the strain did not result in a similar effect. As for the same starter, there were more flavor compounds metabolized in the factory condition than in the laboratory condition, especially esters and alcohols. In brief, the fermentation condition made a greater influence than the fermentation starter for broad bean koji. The differences in micro-organisms and enzymes in the koji play a significant role in the fermentation process of broad bean paste-meju. Therefore, further research about the fermentation process and the quality of broad bean paste-meju fermented by the koji manufactured by PN and HN, respectively, in the laboratory and factory conditions, is required.

## Data availability statement

The datasets presented in this study can be found in online repositories. The names of the repository/repositories and accession number(s) can be found below: https://www.ncbi.nlm.nih.gov/, MN859962.

## Author contributions

HL: conceptualization, methodology, roles and writing—original draft, and review and editing. Z-ML: conceptualization, writing—review and editing, and supervision. W-QD: conceptualization, investigation, data curation, formal analysis, methodology, software, and roles and writing—original draft. Q-SZ: conceptualization, resources, and supervision. GC: funding acquisition and project administration. QL: writing—review and editing. Z-HX: supervision. Y-HM: supervision and writing—review and editing. All authors contributed to the article and approved the submitted version.
