# Supplementary material for: The differences between broad bean koji fermented in laboratory and factory conditions by an efficient Aspergillus oryzae
Source: Front Microbiol. 2023 Mar 22;14:1139406. doi: 10.3389/fmicb.2023.1139406 (PMC10074850; doi:10.3389/fmicb.2023.1139406)
Supplement: Supplementary file 1 [file Data_Sheet_1.doc]

Fig. S1 Koji-making process. A: Koji made in lab; B: Koji made in fac (pilot scale).

Fig. S2 SEM images of the blanched broad beans and broad bean koji.

Fig. S3 Principal component analysis (PCA) of microbial gene for broad bean koji.

Fig. S4 The variation trends of each amino acid.

Fig. S5 The variation trends of organic acids.

Fig. S1 Koji-making process. A: Koji made in lab; B: Koji made in fac (pilot scale)

Fig. S2 SEM images of the blanched broad beans and broad bean kojis. (L-M: blanched broad bean in lab; F-M: blanched broad bean in fac; L-PN: PN koji in lab; F-PN: PN koji in fac; L-HN: HN koji in lab; F-HN: PN koji in fac; S: starch; P: protein)

Fig. S3 Principal component analysis (PCA) of microbial gene for broad bean koji (A: bioplot of bacteria; B: score scatter of bacteria; C: bioplot of fungi; D: score scatter of fungi)


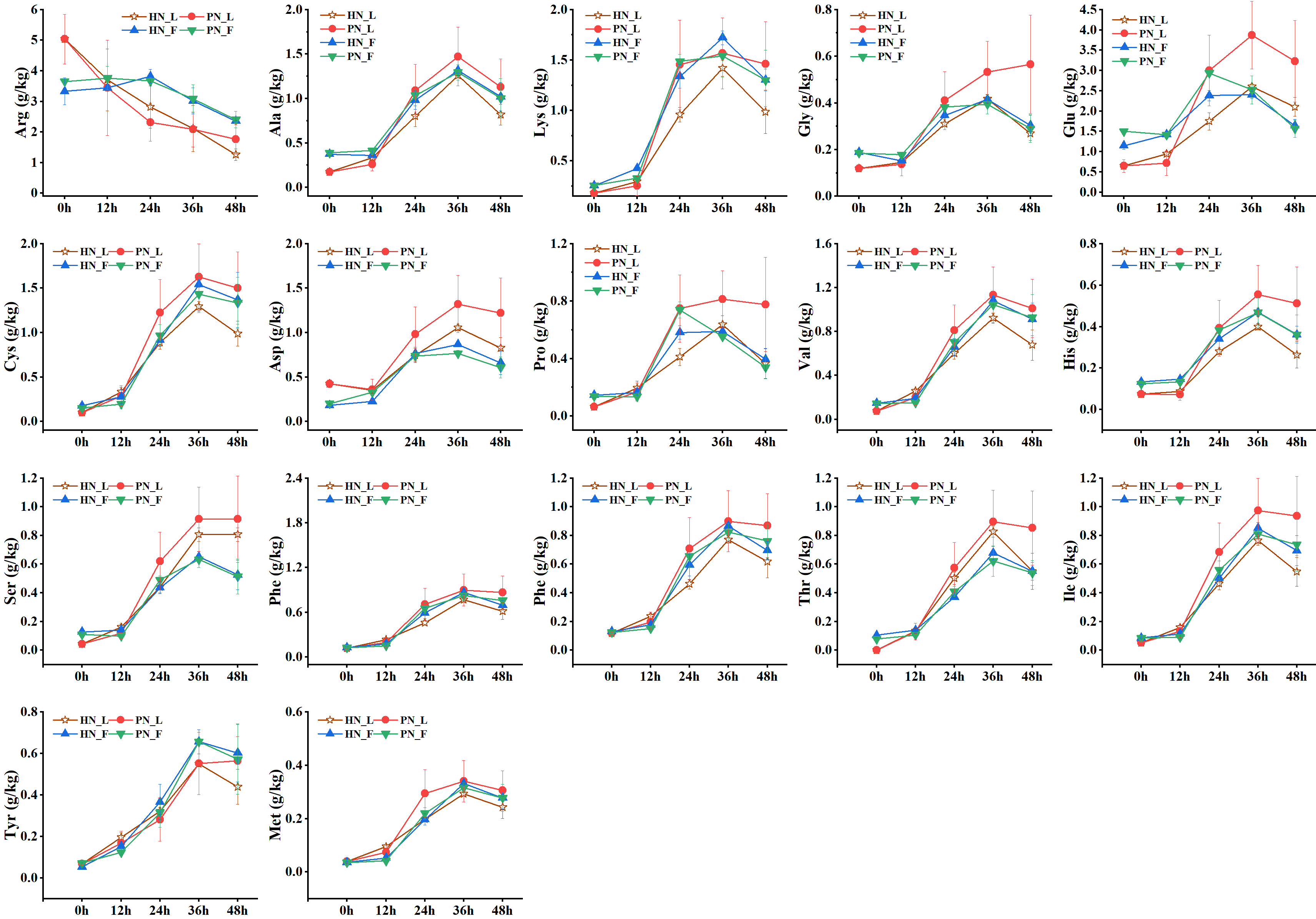
Fig. S4 The variation trends of each amino acids

Fig. S5 The variation trends of organic acids
